# Leukoaraiosis and risk of intracranial hemorrhage and outcome after stroke thrombolysis

**DOI:** 10.1371/journal.pone.0196505

**Published:** 2018-05-01

**Authors:** Chun-Ming Yang, Chien-Ling Hung, Hui-Chen Su, Huey-Juan Lin, Chih-Hung Chen, Chou-Ching Lin, Han-Hwa Hu, Sheng-Hsiang Lin, Pi-Shan Sung

**Affiliations:** 1 Department of Neurology, Chi Mei Medical Center, Tainan, Taiwan; 2 Department of Surgery, National Cheng Kung University Hospital, College of Medicine, National Cheng Kung University, Tainan, Taiwan; 3 Institute of Clinical Medicine, College of Medicine, National Cheng Kung University, Tainan, Taiwan; 4 Department of Neurology, National Cheng Kung University Hospital, College of Medicine, National Cheng Kung University, Tainan, Taiwan; 5 Department of Neurology, School of Medicine, College of Medicine, Taipei Medical University, Taipei, Taiwan; 6 Department of Neurology, Shuang Ho Hospital, Taipei Medical University, Taipei, Taiwan; Monash University, AUSTRALIA

## Abstract

**Background:**

The impact of leukoaraiosis on the risk of symptomatic intracerebral hemorrhage (SICH) after stroke thrombolysis is conflicting, and the data on Asian populations are lacking. Therefore, in this study, we assessed the association between leukoaraiosis and SICH, and the association between leukoaraiosis and the 90-day functional outcome in the Asian population.

**Methods:**

Data were collected from a two-center prospective registry of acute ischemic stroke patients given intravenous tissue plasminogen activator between 2006 and 2014. A total of 614 pretreatment brain CT and 455 posttreatment MRI were retrospectively assessed using two different rating scales for the presence of leukoaraiosis. Outcome measures were the occurrence of SICH with three definitions and any hemorrhage after thrombolysis and functional outcome at 3 months.

**Results:**

Of the 614 patients assessed, 30.3% showed severe leukoaraiosis on the baseline brain CT. The SICH rate was 4.6% - 7.2% based on different definitions, and overall, 24.9% of patients showed any post-tPA hemorrhage. No association was observed between the severity of leukoaraiosis and SICH, regardless of having used different leukoaraiosis rating scales or as assessment using different imaging modalities. However, severe leukoaraiosis was independently associated with poor functional outcome at 3 months (OR 1.96, 95% C1 1.24–3.11, *P* = 0.004) after adjustment for confounders.

**Conclusions:**

Our results showed no association between leukoaraiosis and the risk of SICH. Although the presence of severe leukoaraiosis predicted a poor functional outcome after stroke, IV thrombolysis might not be withheld in acute ischemic stroke patients solely based on the presence of severe leukoaraiosis on pre-thrombolytic CT scans.

## Introduction

Stroke is a major cause of disability and death. The use of intravenous (IV) thrombolysis with recombinant tissue plasminogen activator (tPA) given within 3 hours of onset or within 3–4.5 hours of specific selected strokes in improving the outcome of patients with acute ischemic stroke (AIS) has been well demonstrated [1, 2]. Although IV-tPA is an effective therapy, it also increases the risk of intracerebral hemorrhage (ICH). Symptomatic ICH (SICH) occurs in 2.4% - 10% of patients within 24–36 hours after thrombolysis [3]. Researchers have made efforts to investigate the predictors of post-tPA SICH, including the timing of thrombolysis, tPA dose, patient age, clinical stroke severity, blood pressure before and after tPA treatment, stroke subtype, and imaging features [3, 4]. Potential imaging predictors include early ischemic signs, and stroke lesions covering more than a third of middle cerebral artery territory.

Leukoaraiosis, also known as white matter changes (WMC), is defined as a diffuse, confluent white matter abnormality (with a low density on brain computed tomography [CT] images and hyperintensity on T2-weighted or fluid-attenuated inversion recovery sequences [FLAIR] MRI images) [5]. Although age and hypertension are the strongest risk factors for leukoaraiosis [6, 7], the disease is also commonly associated with other vascular risk factors, such as diabetes, cardiac diseases, or stroke [5]. Leukoaraiosis has been reported to predispose to ICH, commonly located in the basal ganglia and deep cerebral areas [8]. The association between leukoaraiosis and ICH might be attributed to same small vascular pathology induced by chronic hypertension (lipohyalinosis and Charcot-Bouchard aneurysm) [8, 9]. Severe leukoaraiosis may also be associated with an 8.4-fold increased risk of ICH during oral anticoagulation (warfarin) treatment administered for stroke prevention [10].

The association between leukoaraiosis and post-tPA SICH has been reported, but the results were conflicting. Recent meta-analyses have showed that leukoaraiosis may be associated with a 1.5- to 1.9-fold increase in risk for SICH after tPA treatment [11–13] and a 2-fold greater risk of a poor functional outcome after stroke [11]. In previous studies, patient population, rating scales used to determine leukoaraiosis severity, imaging modalities (CT or MRI used to assess leukoaraiosis), and definitions of SICH have been heterogeneous. In addition, most related studies have been performed in Western countries. Thus, the association of leukoaraiosis and SICH after tPA treatment in Asian population needs further investigation.

The present study aimed to explore the association between leukoaraiosis and the risk of post-tPA SICH. In addition, the relationship between leukoaraiosis and functional outcome after tPA treatment was assessed.

## Materials and methods

### Study participants

This study was approved by the Institutional Ethics Review Board of National Cheng Kung University Hospital (NCKUH) and the ethics committee waived the requirement for informed consent for each patient. As participants of the nationwide Taiwan Stroke Registry (TSR) [14], the two medical centers in this study (NCKUH and the Chi-Mei Hospital (CMH)) have maintained prospective stroke registries according to TSR protocol since 2006. The TSR protocol was described previously [14]. In brief, we prospectively enrolled patients who presented to the hospital within 10 days after stroke onset and received CT and/or MRI for the index stroke. Patient characteristics, comprising demographic data, medical history, comorbidities, stroke severity, treatments, hospital course, and complications, are recorded according to a pre-defined system. In the present study, we retrieved the data of patients with AIS receiving IV-tPA treatment within 4.5 hours after onset from 2006 to 2014. IV thrombolysis was administered according to the National Institute of Neurological Disorders and Stroke (NINDS) criteria and Taiwan Stroke Society guidelines [1, 14, 15]. Age over 80 years was not an absolute contraindication. Initial stroke severity was assessed by the National Institute of Health Stroke Scale (NIHSS) [16]. Prior to the tPA treatment, all patients underwent brain CT scans to exclude ICH. Dose of tPA ranged from 0.6 mg/Kg to 0.9 mg/Kg depending on the discretion of the treating physician, as recommended by the 2008 Taiwan Stroke Society Guidelines [15]. After administration of IV-tPA, all patients were admitted to a stroke intensive care unit for observation for at least 24 hours before being transferred to a stroke unit for continued care. All patients had follow-up brain scans (either CT or MRI) between 24–36 hours after thrombolysis, except for four patients who died after admission without any follow-up brain imaging. Additional scans would be obtained in case of clinical deterioration.

### Assessment of leukoaraiosis

The pre-tPA brain CT scans were retrospectively reviewed by a single reader (PS), who was blinded to the clinical data, including the initial NIHSS and post-tPA outcomes. The presence and extent of WMCs were assessed using the modified Van Swieten scale (mVSS) [17–19]. The mVSS is an extension of the Van Swieten scale and grades the anterior and posterior periventricular WMCs for each hemisphere on a three point scales: 0 (no white matter hypodensity), 1 (restricted to the region adjacent to the ventricle), and 2 (confluent white matter hypodensity from ventricle to the gray matter). The total mVSS scores were obtained by summing the two scores in the anterior and posterior head regions of each hemisphere for a total mVSS score of 0 to 8. An mVSS > 4 was defined as severe leukoaraiosis as per the previous description [19].

For further confirming the association between leukoaraiosis and SICH, we assessed the extent of leukoaraiosis again by reviewing available post-tPA brain MRI scans by the same reader (PS) after completing all the pre-tPA CT reading. The extent of leukoaraiosis was determined on the FLAIR or T2-weighted images, and the severity of leukoaraiosis was rated by another rating system: Age-Related White Matter Changes (ARWMC) scales for MRI scans [20]. Five different regions were assessed in the right and left hemispheres separately, including frontal, parieto-occipital, temporal, basal ganglia and infratentorial region (brainstem and cerebellum). The WMCs were graded as absent (score = 0), focal (score = 1), confluent (score = 2), and diffuse on the entire region (score = 3). The basal ganglia lesion was graded as absent (score = 0), 1 focal lesion (≥ 5mm, score = 1), more than 1 focal lesion (score = 2), and confluent lesions (score = 3). We then assessed the association between leukoaraiosis in MRI scans and SICH.

### Outcome

We defined SICH using three different sets of criteria: SICH_NINDS [1], SICH_ECASS-II [21] and SICH_SITS-MOST [22]. The protocol of determining SICH in each patients were ever described in our previous study [23]. In brief, after reviewing the post-tPA CT or MRI scans and clinical neurological status, the classification of SICH was determined by one senior stroke neurologist in each stroke center (NCKUH, CHC; CMH, LHJ). The functional outcome after tPA treatment was evaluated with modified Rankin Scales (mRS) at 3 months (90-day) by telephone interview. We defined a poor functional outcome as mRS score of 3–6.

### Statistical analysis

Categorical variables were presented as numbers (percentage), and continuous variables as means (standard deviation). Patients with no or mild leukoaraiosis (mVSS 0–4) on pre-tPA CT scans were compared with those with severe leukoaraiosis (mVSS > 4 points). We used the Chi-square test to compare categorical variables and the independent T-test to compare continuous variables. The impact of leukoaraiosis on SICH was evaluated by logistic regression analysis. First, we assessed the effect of severe leukoaraiosis (mVSS > 4 points) or any leukoaraiosis (mVSS ≥ 1) in pre-tPA CT scans on SICH. Then we re-evaluated the association between leukoaraiosis in post-tPA MRI scans and SICH by using ARWMC scores as continuous variables. For assessing the intra-rater reliability by one single rater (PS), the kappa statistic was used to compare the scoring of leukoaraiosis between CT and MRI scans for a selected group of 41 patients aged older than 80 years. Logistic regression was then used to assess the relationship between the severe leukoaraiosis (mVSS > 4 points) and the 90-day poor functional outcome (mRS ≥ 3). Potential confounders with P < 0.2 in univariate analysis were included in the multivariate model to examine whether an independent relationship exists between severe leukoaraiosis and 90-day functional outcome.

All analyses were conducted using the statistical software package SAS version 9.4 (SAS Institute Inc., Cary, North Carolina). Statistical significance was set at the *P* < 0.05 level, two-tailed.

## Results and discussion

### Results

**Fig 1:**
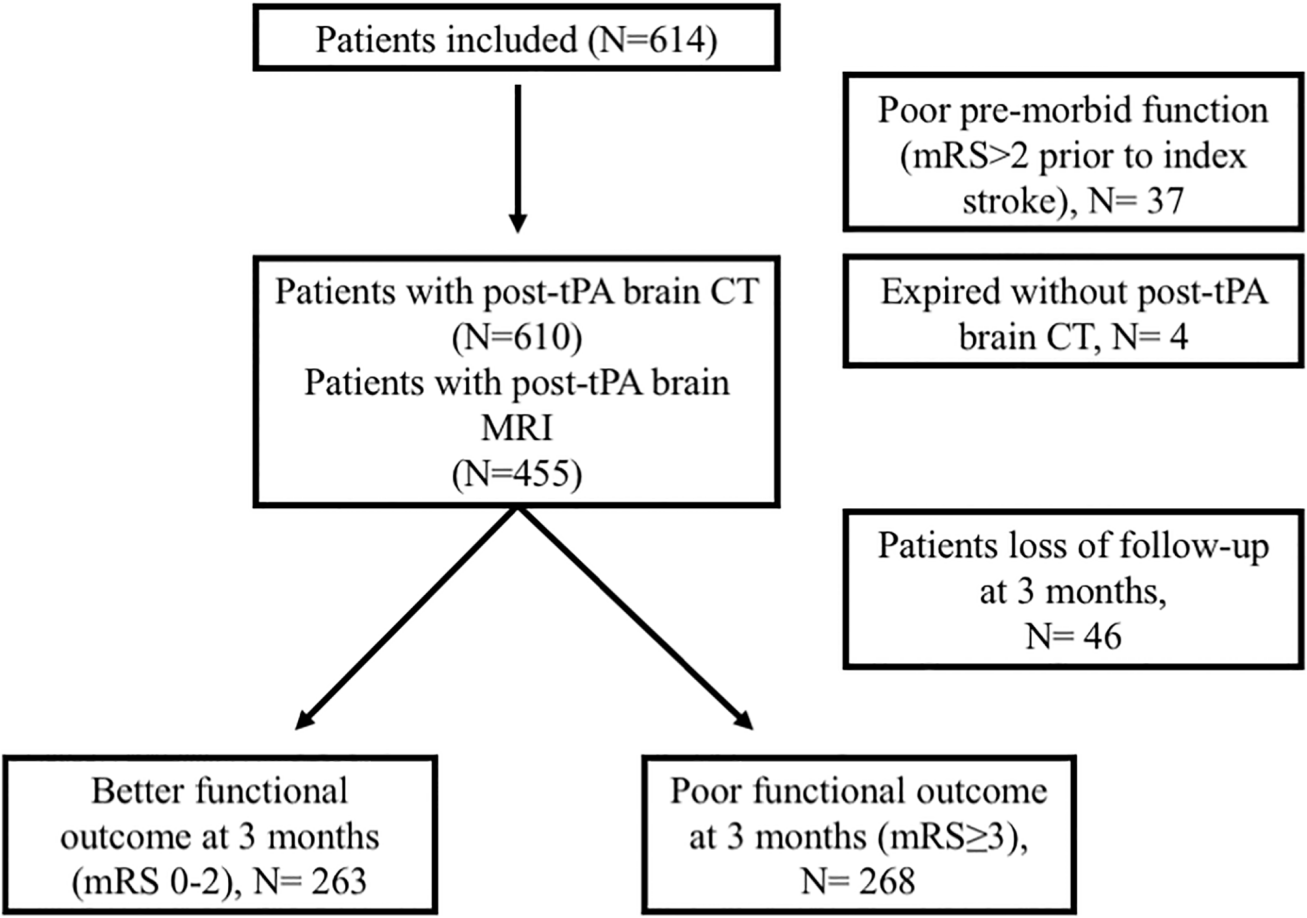
Flow chart of this study. Four patients were excluded in the association analysis of leukoaraiosis and SICH (Expired without post-tPA brain CT). Total 83 patients were excluded (poor pre-morbid function and loss of follow-up at 3 months) in the association analysis of leukoaraiosis and 90-day functional outcome. Note that all 455 patients with post-tPA MRI had post-tPA CT follow-up images, too.

A total of 614 eligible patients with AIS who received IV thrombolysis were identified, comprising 253 patients in CMH and 361 patients in NCKUH (Fig 1). The mean age was 67.4 ± 12.6 years and 61.9% of patients were male. The median NIHSS was 12.5 points. The kappa statistics for the neuroimages of the 41 patients aged older than 80 years ranged from 0.8 to 1.0 for the grading of white matter changes in the four regions examined (the white matter in the left and right frontal and parieto-occipital areas), indicating sufficient agreement of leukoaraiosis severity between CT and MRI.

Among all 614 patients, severe leukoaraiosis, defined as mVSS > 4, was present in 186 patients (30.3%), whereas 246 patients (40.1%) exhibited no leukoaraiosis. Comparisons between the patients with mVSS > 4 and those with mVSS ≤ 4 are presented in Table 1. The patients with severe leukoaraiosis were significantly older than patients with no or mild leukoaraiosis. They also had a smaller percentage of patients with a good pre-stroke functional status (defined as mRS 0–1), hypertension, stroke history and ischemic heart disease than those with no or mild leukoaraiosis. There was no difference in the stroke severity evaluated by the NIHSS, and the prevalence of pre-stroke anti-thrombotic or anti-coagulation therapy between the groups. The mean dose of tPA was similar between the patients with mVSS > 4 and those with mVSS ≤ 4, However, exact body weight was not available before IV-tPA administration in the emergency department. The dose of tPA was depending on the patient’s estimated body weight provided by family. The final dose of tPA may exceed 0.9 mg/Kg in some cases while the exact body weight obtained after admission was lower than the estimated body weight.

**Table 1:** Demographic data and baseline characteristics.

	mVSS ≤ 4 (N = 428)	mVSS > 4 (N = 186)	P values
Age, mean (SD)	64.4 (12.7)	74.1 (9.3)	<0.001
Male, N(%)	273 (63.8)	107 (57.5)	0.14
Good pre-MRS (0–1), N (%)	400 (93.5)	145 (78.0)	<0.001
Vascular risk factors, N(%)			
HTN	311 (72.7)	153 (82.3)	0.01
DM	145 (33.9)	61 (32.8)	0.79
Prior stroke	68 (15.9)	49 (26.3)	0.002
CHF	21 (4.9)	16 (8.6)	0.08
Af	129 (30.1)	56 (30.1)	0.99
IHD	89 (20.9)	52 (28.0)	0.05
Smoking	140 (32.71)	54 (29.0)	0.37
Hyperlipidemia	249 (58.2)	111 (59.7)	0.73
CKD (Cr ≥ 1.5)	60 (14.0)	36 (19.4)	0.09
NIHSS at ER, median (range, IQR)	12.0 (2–38, 10)	13.0 (3–29, 12)	0.18
Prior AP/ AC use, N(%)	132 (30.8)	70 (37.6)	0.10
tPA dose/Kg, mean (SD)	0.86 (0.09)	0.86 (0.09)	0.81

Af: atrial fibrillation; AP: antiplatelet; AC: anticoagulant; CHF: congestive heart failure; CKD: chronic kidney disease; DM: Diabetes mellitus; ER: emergency room; HTN: hypertension; IHD: ischemic heart disease.

The occurrence of any post-tPA hemorrhage and SICH, based on the aforementioned three definitions, are listed in Table 2. There was no significant difference in the risk of SICH between patients with and without severe leukoaraiosis, regardless of the SICH definition used for analysis. The association between any post-tPA hemorrhage and SICH and any leukoaraiosis was also analyzed and is presented in S1 Table. It showed no difference in the risk of hemorrhage between patients with or without any leukoaraiosis (defined as mVSS = 0 vs. mVSS ≥ 1). We further analyzed the association of leukoaraiosis with SICH (using the ECASS-II definition, median value of three SICH rate by different definitions) stratified by different baseline factors. It was noted that age, more severe stroke, prior antithrombotic use or the presence of HTN, DM, prior stroke, ischemic heart disease, chronic kidney disease, or the dose of tPA had no effect on the association of leukoaraiosis and SICH (S2 Table).

**Table 2:** The incidence of SICH and any hemorrhage in patients with different severity of leukoaraiosis and the effect of leukoaraiosis on SICH.

N(%)	Incidence	mVSS ≤ 4 (n = 424*)	mVSS > 4 (n = 186)	OR	95%CI	p
SICH(NINDS)	44(7.2)	29 (6.8)	15 (8.1)	1.20	0.63–2.29	0.59
SICH(ECASS-II)	33(5.4)	24 (5.7)	9 (4.8)	0.85	0.39–1.86	0.68
SICH (SITS-MOST)	28(4.6)	20 (4.7)	8 (4.3)	0.87	0.38–1.99	0.73
Any post-tPA hemorrhage	152(24.9)	107 (25.2)	45 (24.2)	0.93	0.62–1.39	0.71

OR: Odds ratio

*The mVSS of four patients who died during admission without follow-up brain CT were all ≤ 4.

Of the 614 patients, 455 (74%) had post-tPA brain MRI data. The proportion of patients who had any hemorrhage or SICH was lower among patients who underwent post-tPA brain MRI than those who did not (SICH_NINDS: 4.8% vs. 14%; any hemorrhage: 22% vs. 33%). The association between leukoaraiosis in post-tPA MRI scans and SICH still showed a negative correlation between the risk of SICH or any hemorrhage and the total ARWMC scores (Table 3).

**Table 3:** The risk of SICH and any hemorrhage in patients with different levels of severity of leukoaraiosis based on MRI examination (N = 455).

MR ARWMC total scores †	OR	95%CI	P value
SICH(NINDS)	0.95	0.86–1.05	0.30
SICH(ECASSII)	0.94	0.82–1.07	0.32
SICH (SITS-MOST)	0.91	0.77–1.07	0.24
Any post-tPA hemorrhage	0.96	0.91–1.00	0.07

OR: Odds ratio

^†^ ARWMC used as a continuous variable

For analyzing the association between leukoaraiosis and the 90-day functional outcome, we first excluded patients with poor pre-morbid functional status (defined as mRS > 2;n = 37) and also excluded patients with missing follow-up at 3 months (n = 46). Data from 531 patients were analyzed. Univariate analysis showed severe leukoaraiosis predicted a poor 90-day functional outcome (*P* < 0.001). Other potential predictors included older age (*P* = 0.002), female (*P* = 0.03), greater initial stroke severity (*P* < 0.001), DM history (*P* = 0.02), the presence of any post-tPA hemorrhage (*P* < 0.001) or SICH after tPA (*P* < 0.001 in SICH_NINDS, *P* < 0.001 in SICH_ECASS-II, *P* < 0.001 in SICH_SITS-MOST), and lower body weight (*P* = 0.001) (S3 Table). The impact of leukoaraiosis on poor functional outcome at 3 months remained significant after adjustment for potential confounders (OR 1.96, 95% CI 1.24–3.11, *P* = 0.004). Higher initial NIHSS, the presence of baseline DM and lower body weight, and the presence of any post-tPA hemorrhage were also independent predictors for poor functional outcome at 3 months (Table 4).

**Table 4:** Independent predictors of poor functional outcome at 3 months determined using multivariate analysis.

	OR^a^	95% CI	P values
Age	1.00	0.98–1.02	0.86
Male	1.09	0.70–1.70	0.70
NIHSS at ER	1.13	1.09–1.17	<0.001
DM	1.86	1.22–2.83	0.004
CHF	1.32	0.56–3.11	0.52
Any post-tPA hemorrhage	3.03	1.84–4.99	<0.001
CT_mVSS > 4	1.96	1.24–3.11	0.004
Body weight	0.98	0.96–0.99	0.01

^a^ Logistic regression

ER: emergency room; DM: Diabetes mellitus; CHF: congestive heart failure.

### Discussion

The main finding of our results is that the presence of severe leukoaraiosis on pre-tPA brain CT scans was not associated with the risk of any hemorrhage or SICH after tPA treatment. This lack of association was further confirmed by using different leukoaraiosis assessment scales (ARWMC) on post-tPA MRI scans, which are usually considered more sensitive for detecting WMCs. For comparison with previous reports, we tested the three commonly used definitions of SICH. Although there was no association between leukoaraiosis and SICH, the presence of severe WMCs increased the risk of poor functional outcome at 3 months after acute stroke, and leukoaraiosis remained an independent predictor after adjustment for confounding factors.

The SICH rate in our study, 7.2% per the NINDS and 5.4% per the ECASS-II definition, was similar to the mean SICH rate in clinical trials (7.61% in NINDS and 5.61% in ECASS) [24] and previous studies conducted in Taiwan (7.8–8.2% per the NINDS definition and 5.4% per the ECASS-II definition) [14, 25]. However, the SICH rate per the SITS-MOST definition (4.6%) was slightly higher than the mean SICH rate from other studies (3.25%) [24]. No difference existed in the mean age of study participants, initial stroke severity assessed by NIHSS and tPA dosage compared with that in the SITS-MOST trial and SITS-ISTR studies [22, 26, 27]. The GWTG -Stroke program in the Unites States [28] has indicated that Asians may have a higher risk of post-tPA SICH rate (per the NINDS definition) than the White, Black, and Hispanic populations. However, the SICH rate per the NINDS definition in our study appeared to be similar to the SICH rate in other trials conducted in western countries.

In our study, the percentage of patients with severe leukoaraiosis (30.3%) was higher than that found in previous studies (15% - 25%) [18, 19, 29–33]. Furthermore, patients with severe leukoaraiosis (mVSS >4) were younger in our cohort than those with similarly severe leukoaraiosis in other studies (mean age 71 years vs. 75–81 years). The higher prevalence of severe leukoaraiosis in our cohort may be explained by the higher percentage of pre-existing HTN in our cohorts (82%, which was similar to the 79.2% reported by GWTG-Taiwan [14]) compared with the 56% - 79% prevalences of pre-existing HTN among patients in previous studies on leukoaraiosis and post-tPA hemorrhage [18, 19, 30, 31]. Another explanation may be a higher proportion of small vessel disease and intracranial large artery stenosis in Taiwanese and East Asian populations as compared with people in western countries [14, 34, 35], these two stroke subtypes being associated with leukoaraiosis [5, 36]. Any genetic susceptibility for various ethnicities to develop leukoaraiosis is still undetermined.

Our data showed negative results regarding the association between leukoaraiosis and the risk of SICH or any post-tPA hemorrhage. Very few studies regarding this issue were conducted in Asian countries [30], and conflicting results were shown in previous studies performed in Western populations [18, 19, 29, 31–33]. Variations in rating tools, imaging modalities, and various definitions of SICH used in other studies made comparison difficult. To further confirm the relationship between leukoaraiosis and SICH, two different rating scales were applied to grade images obtained using two imaging modalities, and three different definitions of SICH were used. All results showed a lack of association between leukoaraiosis and SICH or any hemorrhage. By contrast, previous research demonstrated a positive and significant association between leukoaraiosis and SICH only in univariate analysis, but the association became nonsignificant after adjustment for other confounders [31]. Additionally, studies showing that leukoaraiosis increases the risk of SICH have all been conducted in the Western populations. Thus, ethnic differences may be another explanation for the difference between our results and those of previous studies.

As with most previous studies and meta-analysis, our findings showed that leukoaraiosis may increase the risk of poor functional outcome after stroke and was a predictor independent of age, stroke severity, and the presence of any hemorrhage. The potential mechanisms leading to poor functional recovery include that leukoaraiosis may increase the risk of recurrent stroke, dementia and death [37, 38], may be associated with reduced cerebral resting blood flow [39], and may impair the brain connectivity system that is important for recovery from ischemic damage [40].

Our study had several limitations. The first was potential selection bias. Because of the selection of IV-tPA, patients prone to bleeding tendency such as those of very old age; patients with poorly controlled blood pressure; and patients with high pre-morbid mRS scores may have been excluded. Therefore, we might have underestimated the association between leukoaraiosis and risk of ICH, and thus the generalizability of our study findings might be limited. Additionally, patients with post-tPA SICH or any hemorrhage may not undergo subsequent MRI. Therefore, the proportion of patients who had SICH or any hemorrhage was lower among patients who received MRI than among those who did not. This may lead to underestimation of the effect of leukoaraiosis on the risk of ICH when post-tPA MRI data were used for analysis. A third limitation is that there were overall 7.5% of our patients lost to clinical or telephone interview follow-up at 3 months after stroke, and we used telephone interview in this study to assess the functional outcome. This might potentially underestimate or overestimate the functional recovery. The severity of leukoaraiosis was rated by a single reader, and thus the readings may have been subject to errors. However, the mVSS is a simple and straightforward rating scale. In the CASES study [19], the inter-rater agreement between two readers was near perfect with kappa values ranging from 0.9 to 1.0. In addition, the single reader in our study demonstrated adequate agreement in grading white matter severity. Therefore, we deemed that the inclusion of only one reader did not substantially affect our results. Finally, including only participants in two medical centers in the Southern part of Taiwan might limit the generalizability of our results. However, our patient number was large and we used two imaging modalities (CT and MRI) and various definitions of SICH for re-confirming the association of leukoaraiosis and SICH. Therefore, our results may serve as reliable reference points for comparisons with the results of other studies.

## Conclusion

Our study demonstrated that presence of leukoaraiosis did not increase the risk of SICH after IV thrombolysis. The lack of association did not alter in patients with different baseline risk factors, such as age, stroke severity or different dose of tPA. However, severe leukoaraiosis was associated with poor functional outcomes at 3 months. Despite the potential risk of poorer functional outcome, IV thrombolysis might not be withheld in patients with AIS solely on the basis of the presence of severe leukoaraiosis on pre-thrombolytic CT images.

## Supporting information

S1 Table The incidence of SICH and any hemorrhage in patients with or without any leukoaraiosis and the effect of leukoaraiosis on SICH. (DOCX)

S2 Table The risk of SICH in patients with different levels of severity of leukoaraiosis stratified using different baseline risk factors. (DOCX)

S3 Table The potential predictors of poor functional outcome at 3 months determined using univariate analysis. (DOCX)
