# Supplementary material for: Leukoaraiosis and risk of intracranial hemorrhage and outcome after stroke thrombolysis
Source: PLoS One. 2018 May 1;13(5):e0196505. doi: 10.1371/journal.pone.0196505 (PMC5929505; doi:10.1371/journal.pone.0196505)
Supplement: S1 Table — (DOCX) [file pone.0196505.s001.docx]

**S1 Table. The incidence of SICH and any hemorrhage in patients with or without any leukoaraiosis and the effect of leukoaraiosis on SICH.**

| **N(%)**  **(N=610)** | **Incidence** | **mVSS = 0 (n=243 †)** |  | **mVSS ≥ 1 (n=367*)** | **OR** | **95%CI** | **p** |
| --- | --- | --- | --- | --- | --- | --- | --- |
| SICH(NINDS) | 44(7.2) | 17(7.0) |  | 27(7.4) | 1.06 | 0.56-  1.98 | 0.87 |
| SICH(ECASS-II) | 33(5.4) | 15(6.2) |  | 18(4.9) | 0.78 | 0.39-  1.59 | 0.50 |
| SICH (SITS-MOST) | 28(4.6) | 13(5.4) | 15 | 15(4.1) | 0.75 | 0.35-  1.61 | 0.47 |
| Any post-tPA hemorrhage | 152(24.9) | 62(25.5) | 9 | 90(24.5) | 0.95 | 0.65-  1.38 | 0.78 |

**OR: Odds ratio**

**† Three patients with mVSS = 0 died during admission without follow-up brain CT and MRI.**

***One patients with mVSS ≥ 1 died during admission without follow-up brain CT or MRI.**
