# Supplementary material for: Leukoaraiosis and risk of intracranial hemorrhage and outcome after stroke thrombolysis
Source: PLoS One. 2018 May 1;13(5):e0196505. doi: 10.1371/journal.pone.0196505 (PMC5929505; doi:10.1371/journal.pone.0196505)
Supplement: S2 Table — (DOCX) [file pone.0196505.s002.docx]

**S2 Table. The risk of SICH in patients with different levels of severity of leukoaraiosis stratified using different baseline risk factors.**

| **N=610** | **SICH Incidence (%)** | | **OR** | **95%CI** | **p** |
| --- | --- | --- | --- | --- | --- |
|  | mVSS ≤ 4 | mVSS > 4 |  |  |  |
| **Age** |  |  |  |  |  |
| ≥ 80 (n=94) | 5.26 (2/38) | 5.36 (3/56) | 1.02 | 0.16 - 6.41 | 0.98 |
| 60-80 (n=364) | 5.62 (14/249) | 4.35 (5/115) | 0.76 | 0.27 – 2.17 | 0.61 |
| < 60 (n=152) | 5.84 (8/137) | 6.67 (1/15) | 1.15 | 0.13 – 9.90 | 0.90 |
| **DM** |  |  |  |  |  |
| Yes (n=205) | 9.02 (13/144) | 4.92 (3/61) | 0.52 | 0.14 – 1.90 | 0.32 |
| No (n=405) | 3.93 (11/280) | 5.13 (6/117) | 1.23 | 0.45 - 3.41 | 0.69 |
| **HTN** |  |  |  |  |  |
| Yes (n=461) | 5.84 (18/308) | 3.27 (5/153) | 0.54 | 0.20 – 1.50 | 0.24 |
| No (n=149) | 5.17 (6/116) | 12.12 (4/33) | 2.53 | 0.67 – 9.56 | 0.17 |
| **Pre-stroke functional status** |  |  |  |  |  |
| Pre-mRS 0-1  (n=541) | 5.81 (23/396) | 3.45 (5/145) | 0.58 | 0.22 – 1.55 | 0.28 |
| Pre-mRS ≥2  (n=69) | 3.57 (1/28) | 9.76 (4/41) | 2.92 | 0.31 – 27.60 | 0.35 |
| **Prior stroke** |  |  |  |  |  |
| Yes (n=117) | 2.94 (2/68) | 2.04 (1/49) | 0.69 | 0.06 – 7.80 | 0.76 |
| No (n=493) | 6.18 (22/356) | 5.84 (8/137) | 0.94 | 0.41 – 2.17 | 0.89 |
| **IHD** |  |  |  |  |  |
| Yes (n=141) | 6.74 (6/89) | 5.77 (3/52) | 0.85 | 0.20 – 3.54 | 0.82 |
| No (n=469) | 5.37 (18/335) | 4.48 (6/134) | 0.83 | 0.32 – 2.13 | 0.69 |
| **CKD** |  |  |  |  |  |
| Yes (n=95) | 6.78 (4/59) | 5.56 (2/36) | 0.81 | 0.14 – 4.66 | 0.81 |
| No (n=515) | 5.48 (20/365) | 4.67 (7/150) | 0.84 | 0.35 – 2.04 | 0.71 |
| **NIHSS at ER** |  |  |  |  |  |
| NIHSS ≤15  (n=385) | 4.29 (12/280) | 5.71 (6/105) | 1.35 | 0.50 – 3.70 | 0.56 |
| NIHSS>15  (n=225) | 8.33 (12/144) | 3.70 (3/81) | 0.42 | 0.12 – 1.55 | 0.19 |
| **Prior AC/AP use** |  |  |  |  |  |
| Yes (n=202) | 4.55 (6/132) | 5.71 (4/70) | 1.27 | 0.35 – 4.67 | 0.72 |
| No (n=408) | 6.16 (18/292) | 4.31 (5/116) | 0.69 | 0.25 – 1.89 | 0.47 |
| **tPA Dose (mg/Kg)** |  |  |  |  |  |
| ≤0.7 (n=44) | 2(6.1) | 2(18.2) | 3.45 | 0.42-28.01 | 0.25 |
| 0.7~0.9 (n=287) | 12(6.4) | 5(5.1) | 0.79 | 0.27-2.32 | 0.67 |
| >0.9 (n=279) | 10(5.0) | 2(2.6) | 0.51 | 0.11-2.39 | 0.39 |

**SICH based on ECASS-II definition**

**IHD: Ischemic heart disease; AC: anticoagulant; AP: antiplatelet**

**OR: Odds ratio**
