# Supplementary material for: Leukoaraiosis and risk of intracranial hemorrhage and outcome after stroke thrombolysis
Source: PLoS One. 2018 May 1;13(5):e0196505. doi: 10.1371/journal.pone.0196505 (PMC5929505; doi:10.1371/journal.pone.0196505)
Supplement: S3 Table — (DOCX) [file pone.0196505.s003.docx]

**S3 Table. The potential predictors of poor functional outcome at 3 months determined using univariate analysis**

| **Variable**  **(N= 531)** | **Better mRS (0-2) (N=263)** | **Poor mRS (≥3) (N=268)** | **P values** |
| --- | --- | --- | --- |
| Age , mean (SD) | 64.6 (11.8) | 68.3 (12.7) | 0.002 |
| Male (N, %) | 180 (68.4) | 159 (59.3) | 0.03 |
| NIHSS at ER, median (IQR) | 9.0 (6.0) | 16.00 (10.00) | <0.001 |
| Vascular risk factors (N,%) |  |  |  |
| HTN | 193 (73.4) | 205 (76.5) | 0.41 |
| DM | 76 (28.9) | 104 (38.8) | 0.02 |
| Hyperlipidemia | 156 (59.3) | 155 (57.8) | 0.73 |
| Af | 72 (27.4) | 84 (31.3) | 0.32 |
| Prior stroke | 51 (19.4) | 41 (15.3) | 0.21 |
| IHD | 64 (24.3) | 63 (23.5) | 0.82 |
| CHF | 10 (3.8) | 20 (7.5) | 0.07 |
| Smoking | 95 (36.1) | 83 (31.0) | 0.21 |
| CKD (>1.5) | 38 (14.5) | 44 (16.4) | 0.53 |
| SICH(NINDS) (N, %) | 1 (0.4) | 36 (13.4) | <0.001 |
| SICH(ECASS_II) (N, %) | 0 (0.0) | 27 (10.1) | <0.001 |
| SICH (SITS-MOST) (N, %) | 0 (0.0) | 24 (9.0) | <0.001 |
| Any post-tPA hemorrhage | 30 (11.4) | 94 (35.1) | <0.001 |
| CT_mVSS>4 (N,%) | 55 (21.0) | 91 (34.0) | <0.001 |
| BW, mean (SD) | 66.8 (11.8) | 63.3 (12.52) | 0.001 |

**Af: atrial fibrillation; BW: body weight; CHF: congestive heart failure; CKD: chronic kidney disease; DM: Diabetes mellitus; ER: emergency room; HTN: hypertension; IHD: ischemic heart disease; OR: Odds ratio.**
